# Differential impact of Tafenoquine and Primaquine on *Plasmodium vivax* recurrence: the role of *CYP2D6* variants

**DOI:** 10.1128/aac.01797-25

**Published:** 2026-04-27

**Authors:** Vanessa Karina Castro Godinho, Victor Irungu Mwangi, Manuela Crispim Morais, Adriana Patricia Brelaz Lopes, Jessica Rafaela dos Santos Alves, Marcelo Augusto Mota Brito, Marcus Vinicius Guimarães de Lacerda, Fabio Trindade Maranhão Costa, Anne Cristine Gomes de Almeida, Gisely Cardoso de Melo

**Affiliations:** 1 Programa de Pós-graduação em Medicina Tropical da Universidade do Estado do Amazonas (PPGMT-UEA), Manaus, Brazil; 2 Fundação de Medicina Tropical Doutor Heitor Vieira Dourado (FMT-HVD) 200241, Manaus, Brazil; 3 Universidade Estadual de Campinas (UNICAMP) 28132, Campinas, Brazil; 4 Instituto Leonidas e Maria Deane (ILMD) 560875 , Manaus, Brazil; 5 Programa de Pós-graduação em Ciências Aplicadas a Hematologia da Universidade do Estado do Amazonas (PPGH-UEA), Manaus, Brazil; The Children's Hospital of Philadelphia, Philadelphia, Pennsylvania, USA

**Keywords:** *Plasmodium vivax*, relapses, tafenoquine, *CYP2D6*, poor metabolizer, genetic variability

## Abstract

Malaria, particularly by *Plasmodium vivax*, remains a significant public health challenge in endemic regions. *P. vivax* has the dormant liver-stage hypnozoites that can reactivate, causing relapses, even after the initial blood-stage infection has been cleared. The prevention of these relapses hinges on the use of 8-aminoquinolines such as primaquine (PQ) and tafenoquine (TQ). However, the efficacy of these treatments is associated with Cytochrome P450 2D6 (*CYP2D6*) activity, which influences drug metabolism. This case-control study aimed to evaluate the relationship between *CYP2D6* activity and the efficacy of TQ and PQ in preventing *P. vivax* relapses. Patients with *P. vivax* malaria were included in a nested case-control cohort study conducted at the Fundação de Medicina Tropical Dr. Heitor Vieira Dourado (Manaus, AM, Brazil). Participants were classified as cases (recurrent malaria within 180 days) or controls (patients without recurrence). They were treated with PQ or TQ. Epidemiological data were collected from participants, and blood samples were obtained for *CYP2D6* genotyping by real-time PCR. The results showed that *CYP2D6* polymorphisms did not affect TQ efficacy (*P* = 0.172). However, participants treated with PQ had a higher risk of recurrence until six months (*P* = 0.016). Those with a predicted intermediate or poor metabolizer phenotype (gIM + gPM) were more likely to experience recurrence compared to normal metabolizers (gNM) (*P* = 0.036). Our findings indicate that *CYP2D6* does not impact the efficacy of TQ, whereas *P. vivax* recurrence is associated with predicted metabolic phenotype, with increased risk among patients treated with PQ, suggesting that *CYP2D6* variability influences treatment response, particularly in intermediate/poor metabolizers.

## INTRODUCTION

Malaria caused by *Plasmodium vivax* remains a major obstacle to disease control and elimination efforts, primarily due to the parasite’s ability to form dormant liver-stage hypnozoites, which can reactivate weeks or months after the initial infection (1, 2). Relapses triggered by hypnozoite activation substantially contribute to ongoing transmission and increased morbidity in endemic regions (3, 4).

The elimination of *P. vivax* hypnozoites relies on the use of 8-aminoquinoline drugs, particularly primaquine (PQ) and, most recently, tafenoquine (TQ) (5, 6). Currently, the World Health Organization (WHO) recommends treatment with a 3-day course of chloroquine (CQ) plus a 7-day or 14-day course of PQ to target the hypnozoite reservoir in the liver and prevent relapses. In 2018, TQ was approved by the U.S. Food and Drug Administration (FDA) and recommended by the WHO as a single-dose (300 mg) radical cure regimen to replace PQ (1). In Brazil, the administration of TQ requires prior screening of the patient’s glucose-6-phosphate dehydrogenase (G6PD) activity, and their use is only recommended for individuals with ≥70% enzyme activity to avoid drug-induced hemolysis (7).

The cytochrome P450 2D6 (CYP2D6) gene encodes a hepatic enzyme responsible for metabolizing approximately 20–30% of all clinically prescribed drugs (8). Over 160 allelic variants have been identified, leading to inter-individual variability in metabolic capacity and determining their therapeutic efficacy. These variants are grouped by function and determine phenotypes classified as ultra-rapid (UM), normal (NM), intermediate (IM), or poor metabolizers (PM) (9, 10). This variability influences the efficacy of PQ, which requires bioactivation by the CYP2D6 enzyme (11–13).

Genetic polymorphisms in the (*CYP2D6*) gene can significantly influence the metabolism of antimalarial drugs that depend on this enzymatic pathway (14). Alleles associated with reduced or null enzyme functions have been linked to intermediate or poor metabolizer phenotypes, which have been implicated in treatment failure and increased risk of *P. vivax* relapse (15–17). In contrast, the role of *CYP2D6* in the bioactivation of TQ remains unclear.

Animal models suggest that *CYP2D6* activity is necessary for the liver-stage efficacy of TQ, indicating potential metabolic activation through oxidative pathways (18, 19). However, clinical studies conducted in Australian populations have not demonstrated a significant association between *CYP2D6* poor metabolizer genotypes and the anti-relapse efficacy of TQ (20). St Jean et al. (21) found no association between CYP2D6 metabolic phenotype and the efficacy of TQ, while Llanos-Cuentas et al. (6) also reported no correlation between enzyme activity scores (AS) and the frequency of *P. vivax* recurrences following radical cure with TQ (6, 21).

While the influence of *CYP2D6* genetic variability on PQ treatment outcomes is well established, there is limited evidence regarding its impact on TQ efficacy, particularly in endemic populations such as those in the Brazilian Amazon. Therefore, this study aimed to evaluate the association between *CYP2D6* genetic variants and the risk of *P. vivax* recurrence in patients treated with TQ or PQ in a *Plasmodium vivax* malaria endemic setting.

## MATERIALS AND METHODS

### Study design

This was an analytical case-control study nested within a cohort.

### Study population

Patients with *P*. *vivax* malaria who attended the Fundação de Medicina Tropical Dr. Heitor Vieira Dourado (FMT-HVD), a leading institution for tropical and infectious diseases in Manaus, AM, Brazil, were enrolled in the study between August 2022 and August 2023. The inclusion criteria were: aged over 18 years, either sex, confirmed *P*. *vivax* malaria diagnosis by Giemsa-stained thick blood smear, non-pregnant women, no use of antimalarials in the last 30 days, and no signs of severe malaria or mixed infection.

### Interventions

Participants received a treatment regimen containing an anti-hypnozoite together with CQ for 3 days, with a decreasing dose (10 mg/kg on the 1st day, and 7.5 mg/kg on the 2nd and 3rd days), plus PQ (0.5 mg/kg/day for 7 days) or TQ (single 300 mg dose). These participants also underwent clinical and parasitological follow-up at FMT-HVD up to 90 days post-infection (D0, D28, D42, D60, D90), with an additional 90 days (D180) in which new episodes of malaria were tracked using the Malaria Epidemiological Surveillance Information System (SIVEP-malaria) (22) for the detection of recurrences. Information regarding previous malaria episodes was collected through patient self-reporting at the time of enrollment, considering the entire period of their life.

Cases were defined as individuals treated with PQ or TQ who experienced at least one recurrence, that is, *P. vivax* infection between 28 to 180 days after the first episode, detectable by optical microscopy. Controls were defined as individuals who did not experience any recurrence of *P. vivax* infection during the same follow-up period after treatment with the same therapeutic regimen.

### Sample collection

Peripheral blood samples were collected from participants during in-person visits (D0, D28, D42, D60, and D90) and stored in 4 mL EDTA tubes. Approximately 500 μL of whole blood was used for the subsequent DNA extraction and *CYP2D6* genotyping, as previously described by our group. All participants were matched by ethnicity, sex, and age in a 1:1 ratio, ensuring no significant differences between case and control groups.

### CYP2D6 allelic type and activity score

Genomic DNA was extracted from whole blood samples using the QIAamp Blood Mini Kit (Qiagen, Hilden, Germany), according to the manufacturer’s instructions. *CYP2D6* genotyping was performed using TaqMan assays (Thermo Fisher Scientific, South San Francisco, CA, USA) and on the 7500 Fast Real-Time PCR System (v2.3 software; Applied Biosystems, Foster City, CA, USA) to detect single nucleotide polymorphisms (SNPs). The selection of *CYP2D6* alleles was based on three complementary criteria: documented allele frequency in the Brazilian population, particularly in the Northern region and in Manaus; known functional impact on CYP2D6 enzymatic activity; and clinical relevance in pharmacogenetic studies evaluating PQ response (16, 23, 24). These variants include alleles associated with normal, reduced, and null enzyme activity, allowing accurate inference of predicted metabolizer phenotypes. Specific TaqMan probes were used for each SNP assay, and the corresponding dbSNP identifiers for each probe are listed in Table 1.

**TABLE 1:** Polymorphisms and allelic variants of the *CYP2D6* gene (40)

Variants	Polymorphisms	dbSNP ID	Gene effect	Enzymatic function Phenotype
*1	Reference allele	–^*a*^	–	Normal
*2	4180G>C, −1584C>G, 2850C>T	rs1135840, rs1080985, rs16947	Substitutions (C/G, G/C, A/G)	Normal
*34	2850C>T	rs16947	Substitutions (A/G)	Normal
*35	−1584C>G, 31G>A, 2850C>T, 4180G>C	rs1080985, rs769258, rs16947, rs1135840	Substitutions (G/C, C/T, A/G, C/G)	Normal
*39	4180G>C	rs1135840	Substitutions (C/G)	Normal
*9	2615_2617delAAG	rs5030656	Insertion/Deletion (CTT/-)	Decreased
*10	100C>T, 4180G>C	rs1065852, rs1135840	Substitutions (A/G, C/G)	Decreased
*17	1023C>T, 2850C>T, 4180G>C	rs28371706, rs16947, rs1135840	Substitutions (G/A, A/G, C/G)	Decreased
*29	2850C>T, 3183G>A, 4180G>C	rs16947, rs59421388, rs1135840	Substitutions (A/G, C/T, C/G)	Decreased
*41	2850C>T, 4180G>C, 2988G>A	rs16947, rs1135840, rs28371725	Substitutions (A/G, C/G, C/T)	Decreased
*3	2549delA	rs35742686	Insertion/Deletion (T/-)	Null
*4	100C>T, 1846G>A, 4180G>C	rs1065852, rs3892097, rs1135840	Substitutions (A/G, C/T, C/G)	Null
*5	Gene deletion	–	Deletion	Null
*_XN	Gene amplification	–	Amplification	Allele dependent

^*a*^ –, no change in the gene because it is a reference allele.

The *CYP2D6* gene SNP genotyping reactions were conducted in a final volume of 5 µL, containing 2.5 µL of TaqMan Universal PCR Master Mix (Applied Biosystems), 0.25 µL of Genotyping Assay (Applied Biosystems), and 2.25 µL of genomic DNA, following protocol adapted from Silvino et al. (15). The amplification conditions for CYP2D6 SNP genotyping were as follows: pre-PCR (60°C, 30 s); initial denature/enzyme activation (95°C, 10 min); 50 cycles of denaturation (92°C, 15 s) and annealing/extension (60°C, 1 min 30 s); and post-PCR (60°C, 30 sec).

The quantification of copy number variation (CNV) of the *CYP2D6* gene was also performed by real-time PCR. For this analysis, the reactions contained 5.0 µL of TaqMan Genotyping PCR Master Mix (Applied Biosystems), 0.5 µL of TaqMan Copy Number Reference Assay (RNase P, Applied Biosystems), 2.0 µL of nuclease-free water (Invitrogen), and 2.0 µL of DNA (5 ng/mL). The CNV analysis was performed using CopyCaller v2.1 software (Applied Biosystems, CA, USA). The amplification conditions for CNV quantification were as follows: initial denature/enzyme activation (95°C, 10 min); 40 cycles of denaturation (95°C, 15 s); and annealing/extension (60°C, 1 min). Data were interpreted using CopyCaller v2.1 software (Applied Biosystems, California, USA).

*CYP2D6* diplotypes were inferred in the R environment using the HaploStats package, and star (*) allele assignments followed the current nomenclature provided by the Pharmacogene Variation Consortium (PharmVar) (25). Predicted metabolic phenotypes were defined based on Activity Score (AS), where the AS of a genotype is the sum of the values assigned to each allele: *1 (1.0), *2 (1.0), *3 (0.0), *4 (0.0), *9 (0.25), *10 (0.25), *17 (0.5), *29 (0.5), *34 (1.0), *35 (1.0), *39 (1.0), and *41 (0.25). Based on total AS, phenotypes were classified as follows: slow metabolizer (gPM), AS = 0; intermediate metabolizer (gIM), 0 < AS < 1.25; normal metabolizer (gNM), 1.25 ≤ AS ≤ 2.25; and ultrarapid metabolizer (gUM), AS > 2.25, according to the guidelines of the Clinical Pharmacogenetics Implementation Consortium (CPIC) and the Dutch Pharmacogenetics Working Group (DPWG) (10, 25). The specific polymorphisms investigated in this study are listed in Table 1.

### Data analysis

The data analysis focused on calculating the absolute and relative frequencies of *CYP2D6* polymorphisms, star alleles, and predicted metabolic phenotype. Kaplan-Meier survival curves and Cox proportional hazards models were evaluated to assess the influence of genotypes on time to recurrence. Poisson regression was applied to assess the effect of genotypes on the number of recurrences. The data set was subjected to the Mann-Whitney *U* test to assess whether there were significant differences in the distributions. Analyses were conducted using STATA version 15, with significance defined as *P* ≤ 0.05.

## RESULTS

**Fig 1:**
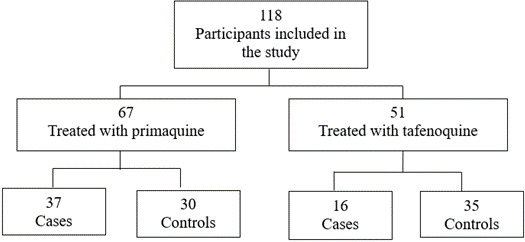
Flowchart of the study design and distribution of the number of participants included in the cohort.

Between August 2022 and August 2023, 118 participants were enrolled in the study and allocated into two treatment arms: primaquine plus chloroquine (PQ + CQ) and tafenoquine plus chloroquine (TQ + CQ). In the PQ group (*n* = 67), 37 patients (55.2%) had at least one recurrence until D180 of follow-up, while 30 (44.8%) remained recurrence-free. In the TQ group (*n* = 51), 16 patients (31.4%) had a clinical recurrence, whereas 35 (68.6%) did not present any new episodes during the follow-up period (Fig. 1).

There were no significant differences observed between cases and controls regarding demographic variables (Table 2). Regarding sex, 75.5% of cases and 75.4% of controls were male. The majority of participants self-identified as mixed race, accounting for 92.5% of cases and 95.4% of controls. The mean age was 42 years for cases (95% CI: 31.0–50.1) and 43 years for controls (95% CI: 34.0–50.0), with no statistically significant difference between the groups (*P* = 0.884; Table 2).

**TABLE 2:** Baseline epidemiological characteristics of participants

	Total = 118	Cases = 53	Controls = 65	*P*-value^*a*^
Sex				0.991
Female	29/118 (24.6%)	13/53 (24.5%)	16/65 (24.6%)	
Male	89/118 (75.4%)	40/53 (75.5%)	49/65 (75.4%)	
Ethnicity				0.220
East Asian	1/118 (0.8%)	1/53 (1.9%)	0/65 (0.0%)	
White	4/118 (3.4%)	1/53 (1.9%)	3/65 (4.6%)	
Mixed race	111/118 (94.1%)	49/53 (92.5%)	62/65 (95.4%)	
Black	2/118 (1.7%)	2/53 (3.8%)	0/65 (0.0%)	
Age in years, median (IQR)	42.5 (32.0–50.0)	42.0 (31.0–51.0)	43.0 (34.0–50.0)	0.884
Parasite density, median (IQR)	4,657.75 (1,806.0–8,240.0)	5,174.0 (1,443.0–8,364.0)	4,576.0 (2,190.0–8,190.0)	0.972
Treatments				0.010
TQ + CQ	51/118 (43.2%)	16/53 (30.2%)	35/65 (53.8%)	
PQ + CQ	67/118 (56.8%)	37/53 (69.8%)	30/65 (46.2%)	
Previous episodes of malaria				0.397
1	35/118 (29.7%)	17/53 (32.1%)	18/65 (27.7%)	
2	26/118 (22.0%)	13/53 (24.5%)	13/65 (20.0%)	
3	31/118 (26.3%)	10/53 (18.9%)	21/65 (32.3%)	
4	25/118 (21.2%)	13/53 (24.5%)	12/65 (18.5%)	
Number of recurrences^*b*^				
1 event		40/53 (75.5%)		
>1 event		13/53 (24.5%)		

^*a*^ *t*-test, chi-square test, or Fisher's exact test.

^*b*^ Recurrences defined as a new episode of malaria occurring after D28 until D180.

Nonetheless, participants who received TQ + CQ had a recurrence rate of 30.2%, whereas those treated with PQ + CQ showed a substantially higher recurrence rate of 69.8%. Most participants reported only one previous episode of malaria, and 75.5% experienced a single recurrence during the follow-up period (Table 2).

### *CYP2D6* allelic frequency and predicted phenotype

Allelic analysis of *CYP2D6* showed a predominance of alleles associated with normal enzymatic activity (*1 and *2, at 34.7% and 25.8%, respectively). Alleles related to intermediate/poor metabolizers (*9, *10, *17, and *41) were observed in both groups, ranging between 2.5 and 6.8%, whereas alleles with null function (*3, *4, and *5) were less frequent (0.4–5.9%), with *5 notable for 3.8% gene deletions (Table 3). A relevant finding was the significant difference in the frequency of the *39 allele between groups, being more prevalent in participants treated with PQ (10.4%) compared to those treated with TQ (1.0%; *P* = 0.002).

**TABLE 3:** Allelic frequency and *CYP2D6* predicted phenotype in participants treated with TQ and PQ^*c*^

	Allele	Total		TQ		PQ		*P*-value^*a*^
		*n* = 236	(%)	*n* = 102	(%)	*n* = 134	(%)	
CYP2D6	*1	82	34.7	38	37.3	44	32.8	0.480
	*2	61	25.8	31	30.4	30	22.4	0.164
	*3	1	0.4	1	1.0	0	0.0	0.432
	*4	14	5.9	5	4.9	9	6.7	0.559
	*5	9	3.8	2	2.0	7	5.2	0.306
	*9	6	2.5	3	2.9	3	2.2	>0.999
	*10	6	2.5	3	2.9	3	2.2	>0.999
	*17	9	3.8	3	2.9	6	4.5	0.735
	*29	0	0.0	0	0.0	0	0.0	N/D^*d*^
	*34	6	2.5	3	2.9	3	2.2	>0.999
	*35	10	4.2	7	6.9	3	2.2	0.106
	*39	15	6.4	1	1.0	14	10.4	0.002
	*41	16	6.8	5	4.9	11	8.2	0.317
	ND	1	0.4	0	0.0	1	0.7	>0.999
*CYP2D6* Predicted phenotype^*b*^	gIM + gPM	28	23.7	9	17.7	19	28.4	0.175
	gNM gUM	90	76.3 0	42	82.4 0	48	71.6 0	0.175 0

^*a*^ Value determined by chi-square test.

^*b*^ Normal activity (gNM), Intermediate/poor activity (gIM/gPM), and increased activity (gUM).

^*c*^ *n*, number of chromosomes.

^*d*^ N/D, not determined.

The predicted phenotype was predominantly normal metabolizers (gNM), representing 76.3% of the participants, followed by intermediate/poor metabolizers (gIM + gPM), which accounted for 23.7%. No ultrarapid metabolizers (gUM) were identified in this study (Table 3).

### *CYP2D6* allelic frequency and predicted phenotype by treatment type and recurrence events

Among participants who experienced recurrence until D180, there were distinct allelic frequency patterns as observed between the TQ and PQ treatment groups. In the TQ case group, the *1 and *2 alleles (associated with normal enzymatic activity) were the most frequent at 40.6% and 21.9%, respectively, while in the TQ control group, they were 35.7% and 34.3%, respectively. The intermediate/poor metabolizer alleles *3, *4, and *5 were present at 9.4% and 3.1% in the case group, and 1.4%, 2.9%, and 1.4% in the control group, respectively (Table 4).

**TABLE 4:** Distribution of alleles and predicted CYP2D6 phenotype in participants treated with tafenoquine or primaquine, stratified by recurrence status^*c*^^,^^*d*^

Gene	Allele	Tafenoquine (TQ)				Primaquine (PQ)			
		Total *N* = 102	Case *n* = 32	Control *n* = 70	*P*-value	Total *N* = 134	Case *n* = 74	Control *n* = 60	*P*-value^*a*^
	*1	38 (37.3%)	13 (40.6%)	25 (35.7%)	0.634	44 (32.8%)	23 (31.1%)	21 (35.0%)	0.631
	*2	31 (30.4%)	7 (21.9%)	24 (34.3%)	0.206	30 (22.4%)	12 (16.2%)	18 (30.0%)	0.057
	*3	1 (1.0%)	0 (0.0%)	1 (1.4%)	>0.999	0 (0.0%)	0 (0.0%)	0 (0.0%)	N/D
	*4	5 (4.9%)	3 (9.4%)	2 (2.9%)	0.176	9 (6.7%)	7 (9.5%)	2 (3.3%)	0.187
	*5	2 (2.0%)	1 (3.1%)	1 (1.4%)	0.531	7 (5.2%)	5 (6.8%)	2 (3.3%)	0.459
CYP2D6	*9	3 (2.9%)	2 (6.3%)	1 (1.4%)	0.231	3 (2.2%)	2 (2.7%)	1 (1.7%)	>0.999
	*10	3 (2.9%)	1 (3.1%)	2 (2.9%)	>0.999	3 (2.2%)	1 (1.4%)	2 (3.3%)	0.587
	*17	3 (2.9%)	0 (0.0%)	3 (4.3%)	0.550	6 (4.5%)	4 (5.4%)	2 (3.3%)	0.691
	*34	3 (2.9%)	2 (6.3%)	1 (1.4%)	0.231	3 (2.2%)	1 (1.4%)	2 (3.3%)	0.587
	*35	7 (6.9%)	2 (6.3%)	5 (7.1%)	>0.999	3 (2.2%)	1 (1.4%)	2 (3.3%)	0.587
	*39	1 (1.0%)	0 (0.0%)	1 (1.4%)	>0.999	14 (10.4%)	9 (12.2%)	5 (8.3%)	0.471
	*41	5 (4.9%)	1 (3.1%)	4 (5.7%)	>0.999	11 (8.2%)	8 (10.8%)	3 (5.0%)	0.344
	N/D	0 (0.0%)	0 (0.0%)	0 (0.0%)	N/D	1 (0.7%)	1 (1.4%)	0 (0.0%)	>0.999
CYP2D6 predicted phenotype^*b*^	gIM + gPM	*N* = 51 (%)	*n* = 16 (%)	*n* = 35 (%)		*N* = 67 (%)	*n* = 37 (%)	*n* = 30 (%)	
		9 (17.7%)	4 (25.0%)	5 (14.3%)	0.436	19 (28.4%)	3 (35.1%)	6 (20.0%)	0.172
	gNM	42 (82.4%)	12 (75.0%)	30 (85.7%)	0.436	48 (71.6%)	4 (64.9%)	24 (80.0%)	0.172
	gUM	0	0	0		0	0	0	

^*a*^ Value determined by chi-square test.

^*b*^ Normal activity (gNM), Intermediate/poor activity (gIM/gPM), and increased activity (gUM).

^*c*^ *n*, number of chromosomes.

^*d*^ N/D, not determined.

Concerning the participants treated with PQ, *1 and *2 allele frequencies were 31.1% and 16.2% in the case group, and 35.0% and 30.0% in the control group, respectively. Alleles *4 and *5 were found at 9.5% and 6.8% in the case group, and both at 3.3% in the control group (Table 4). The most frequent phenotype was normal metabolizer, accounting for 82.4% of those treated with TQ and 71.6% of those treated with PQ. No statistically significant difference was found between the treatment groups (*P* = 0.172).

No statistically significant differences were observed in the proportions of *CYP2D6* metabolizer phenotypes (gNM vs gIM/gPM) between participants case and controls (*P* = 0.172). No ultrarapid metabolizers were identified in this study. Additionally, no significant associations were found between genotypes and allele frequencies across the groups (Table 4).

### CYP2D6 metabolizer phenotype and malaria recurrence

**Fig 2:**
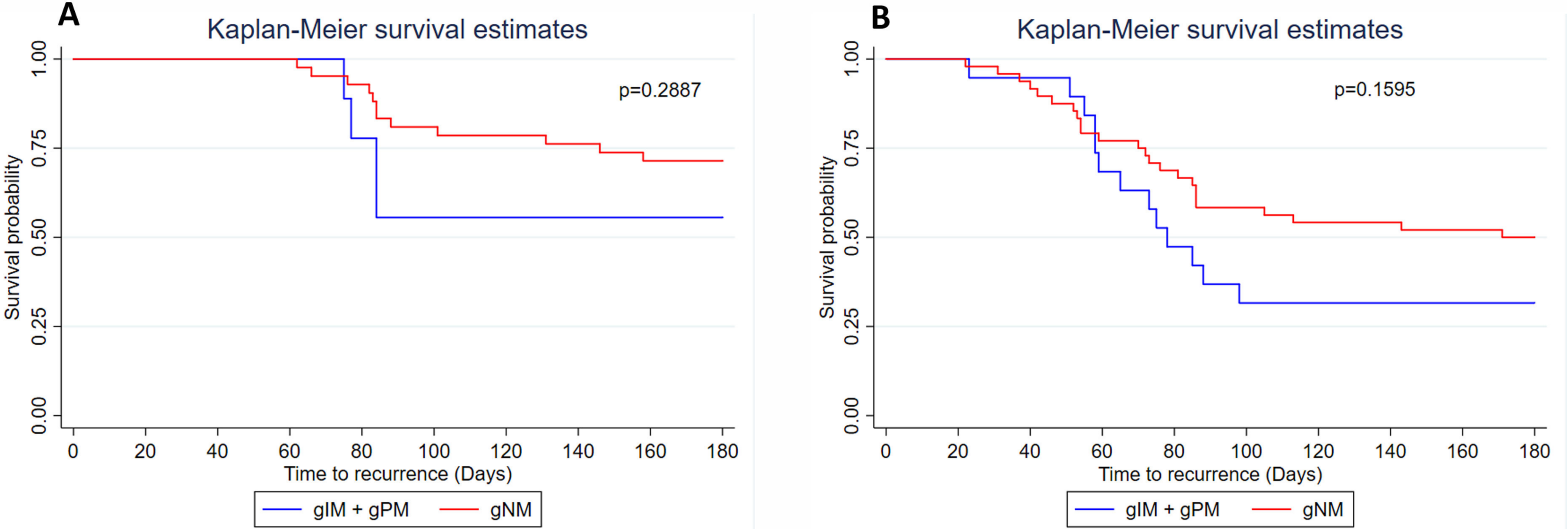
Kaplan-Meier survival curves showing the influence of CYP2D6 activity on time to first recurrence. (**A**) Survival curve over a 180-day period for patients treated with TQ. (**B**) Survival curve over a 180-day period for patients treated with PQ.

The time from participant inclusion to recurrence among TQ group participants ranged between 62 and 158 days, with a median of 84 days (Fig. 2A). Among PQ participants, recurrence occurred between 22 and 171 days, with a median of 70 days (Fig. 2B). A Wilcoxon rank-sum (Mann-Whitney) test revealed that participants in the TQ group showed significantly longer times to first recurrence compared to those in the PQ group (*P* = 0.008). However, there was no statistically significant difference observed in the time to first recurrence between poor/intermediate metabolizer groups and those with normal enzymatic activity.

The relative risk (RR) of recurrence was significantly higher among participants treated with PQ compared to those treated with TQ (1.76 vs 0.57, *P* = 0.016). Furthermore, considering both treatment regimens, participants with intermediate or poor metabolizer phenotypes (gIM/gPM) had a higher risk of recurrent *P*. *vivax* malaria recurrence compared to normal metabolizers (gNM) (1.52 vs 0.66, respectively, *P* = 0.036) (Table 5). The RR analysis considering the separate groups was performed, but there was no significant difference related to the pharmacogenetic profile in each treatment group (for PQ, *P* = 0.3; and for TQ, *P* = 0.1).

**TABLE 5:** Relative risk of malaria recurrence according to predicted CYP2D6 metabolizer phenotype and antimalarial treatment

	Case (*n*)	Control (*n*)	RR (95% CI)^*a*^	*P*-value
gIM + gPM (both treatments)	17	11	1.52 (1.027–2.244)	0.036
gNM (both treatments)	36	54	0.66 (0.446–0.974)	0.036
TQ	16	35	0.57 (0.358–0.899)	0.016
PQ	37	30	1.76 (1.111–2.787)	0.016

^*a*^ Relative risk (RR) with 95% confidence interval (CI).

**Fig 3:**
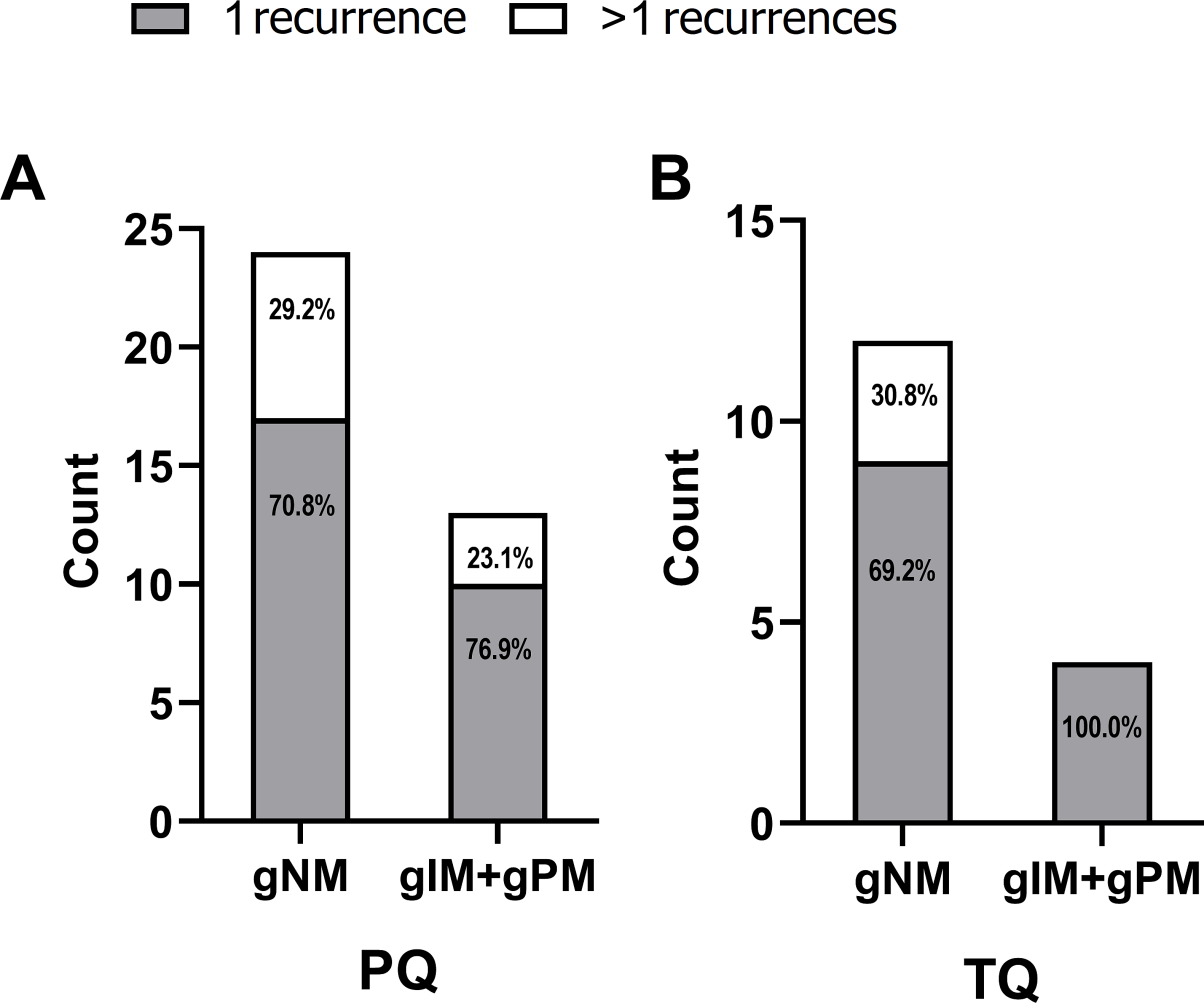
Stacked bar charts showing the distribution of CYP2D6 phenotypes (gNM vs. gIM + gPM) among participants with single and multiple recurrences, stratified by treatment cohort. Panel **A** corresponds to individuals treated with primaquine (PQ), and Panel **B** to those treated with tafenoquine (TQ).

We further investigated the relationship between patient CYP2D6 metabolic phenotype status and the number of malaria recurrences experienced after treatment. Among participants treated with PQ and had the gNM phenotype, 70.8% experienced a single recurrence, while 29.2% had more than one. A similar pattern was observed in those with the intermediate/poor metabolizer (gIM/gPM) phenotype, where 76.9% had one recurrence and 23.1% had more than one. For participants treated with TQ, those with the normal metabolizer (gNM) phenotype, 69.2% experienced a single recurrence, and 30.8% had more than one. However, among the TQ-treated participants with the gIM/gPM phenotype, all experienced a single recurrence (Fig. 3).

## DISCUSSION

To the best of our knowledge, this is the first real-world nested case-control study to evaluate the impact of *CYP2D6* polymorphism on the therapeutic response to TQ and PQ in a Brazilian population endemic for *P. vivax*. Previous investigations into treatment response for *P. vivax* have predominantly focused on the influence of the *CYP2D6* gene on PQ efficacy (11, 13). These studies demonstrated that polymorphisms associated with reduced enzymatic activity (gPM) impaired PQ metabolism and were linked to a higher frequency of malaria recurrences compared to individuals with the gNM phenotype (16, 26). However, evidence regarding the impact of *CYP2D6* variants on TQ metabolism remains limited (27).

PQ is metabolized by the CYP2D6 enzyme, generating phenolic metabolites such as 5-hydroxyprimaquine and 5,6-orthoquinone primaquine, which are associated with oxidative stress generation, hemolytic potential, and parasite killing (28, 29). In contrast, the metabolic pathways involved in TQ activity remain less clearly defined. Experimental studies suggest that CYP enzymes may contribute to the formation of the 5,6-orthoquinone tafenoquine metabolite (5,6-OQTQ) (19). However, pharmacokinetic analyses measuring 5,6-OQTQ in plasma and urine have shown that variability in this metabolite does not appear to correlate with CYP2D6 metabolic phenotype (30). Consistent with this observation, clinical studies have reported no association between CYP2D6 metabolizer status and the risk of *P. vivax* recurrence following TQ treatment (21). These findings suggest that TQ efficacy may rely on metabolic pathways that are less dependent on CYP2D6 activity, potentially explaining the lack of association between *CYP2D6* genotype and anti-relapse efficacy observed in our study.

Similarly, studies in Australian populations reported no significant association between *CYP2D6* genotype or metabolic status and *P. vivax* recurrence following TQ treatment (20). The results from our study align with these findings, as there was no remarkable difference in recurrence rates between gNM and gIM + gPM phenotypes, suggesting that predicted *CYP2D6* metabolic activity does not critically influence the efficacy of TQ in our cohort.

Nevertheless, when evaluating the relative risk of recurrence over 180 days of follow-up, participants treated with PQ had a significantly higher recurrence compared to TQ. This result is consistent with a meta-analysis that demonstrated the superiority of TQ over PQ in preventing *P. vivax* recurrences (31). This effect may be explained, in part, by better adherence to treatment with TQ, administered as a single dose, as well as its prolonged half-life and its presumed lack of dependence on CYP2D6 activity for efficacy (18). Moreover, individuals with gIM/gPM demonstrated an increased risk of recurrence compared to gNM, indicating a potential role of *CYP2D6*-mediated metabolism in PQ efficacy.

Our study included a representative number of participants with the gIM + gPM phenotype, all of whom experienced a single recurrence during the follow-up period. This provides evidence on how the genetic polymorphisms contributing to reduced or absent CYP2D6 enzyme function were associated with patient response to treatment. This observation contributes to the current body of evidence by addressing the gap in previous studies in which such PQ-treated individuals were underrepresented or absent in the TQ treatment arms (24, 31).

Regarding hemolytic potential, evidence from the TRUST study demonstrated that TQ was associated with a lower frequency of hemolytic events compared with PQ when inadvertently administered to individuals with G6PD deficiency (32). One possible explanation for this difference may be related to the distinct metabolic pathways of these 8-aminoquinolines. PQ bioactivation is strongly dependent on CYP2D6 metabolism, generating reactive phenolic metabolites that contribute to oxidative stress and hemolysis (13). A study on the pharmacogenetic profile of patients with *P*. *vivax* malaria in the Brazilian Amazon treated with PQ showed that G6PD-deficient individuals with a CYP2D6 ultra-rapid metabolizer phenotype had increased hepatic and renal markers of hemolysis (33). It is suggested that individuals with ultra-rapid CYP2D6 metabolism generate a greater amount of metabolite, which contributes to an increased risk of hemolytic anemia (34). In contrast, TQ metabolism appears to involve alternative oxidative pathways, including the formation of 5,6-orthoquinone tafenoquine (5,6-OQTQ), which may occur independently of CYP2D6 activity (19, 30). These differences in metabolic activation may result in distinct patterns of reactive oxygen species generation and could partly explain the lower hemolytic risk observed with TQ compared with PQ.

The study participants received treatment as recommended by the Ministry of Health, prescribed according to the institution’s routine and without interference from the research team. Treatment with CQ and PQ/TQ was not supervised by the institution’s health professionals or by the researchers. Previous studies estimated non-adherence to antimalarial treatment in Amazonian locations at around 30% (35, 36). A study conducted in the Amazon region verified the impact of supervised treatment with PQ for the radical cure of *P*. *vivax* malaria (37). Patients undergoing supervised treatment showed a lower risk of recurrence 180 days after the initial episode, when compared to unsupervised treatment (17.9% vs 36.1%, respectively). Similar to the aforementioned study, this study followed a real-world care pattern, allowing for the evaluation of the influence of the pharmacogenetic profile on the frequency of recurrences under uncontrolled conditions. The measure adopted by the research team to try to minimize low adherence to medication was to counsel patients about possible malaria treatments that could be prescribed and the importance of following the treatment to achieve a cure.

The individuals included in the study came from various zones within the city of Manaus, Amazonas, including peri-urban areas, where there is a higher concentration of malaria cases due to proximity to forested areas (38, 39). A portion of the population usually resides in these areas or frequents them for leisure activities in bathing areas or rural properties, or for professional activities such as agriculture and fish farming. A portion of individuals from these areas exhibited episodes of malaria prior to the study period, probably due to reinfections or relapses. Place of residence or prior exposure to malaria was not used as an exclusion criterion in this study. In our study, it was not possible to distinguish the observed recurrences as relapses, since molecular markers, such as microsatellites, were not analyzed.

This study has some limitations that should be acknowledged. First, treatment administration was not supervised, and plasma concentrations of PQ, TQ, or their metabolites were not measured. Although participants received counseling regarding the importance of completing the prescribed antimalarial regimen, incomplete adherence, particularly to the PQ regimen, may have occurred and could have influenced recurrence rates. Second, parasite genotyping was not performed to differentiate relapse, reinfection, or recrudescence. This limitation is particularly relevant because the study was conducted in Manaus, Amazonas, a malaria-endemic region where individuals may reside in or frequently visit peri-urban areas with ongoing transmission, increasing the likelihood of reinfection during follow-up. In addition, this analysis was conducted using a convenience sample of patients with *P. vivax* recurrence after treatment with PQ or TQ, which may have contributed to the relatively high recurrence frequency observed in this cohort. CYP2D6 metabolic phenotypes were inferred from genotype-based activity scores rather than from directly measured enzyme activity. Finally, at the time of data collection, TQ was still in the early stages of implementation in Brazil, which resulted in a smaller number of participants in the TQ treatment arm and may have limited the statistical power to detect modest pharmacogenetic associations.

### Conclusion

In conclusion, for PQ, a higher frequency of recurrence was observed among individuals with the gIM/gPM phenotype compared to those with gNM, indicating that CYP2D6 genetic polymorphisms influenced treatment response. However, TQ did not appear to be significantly affected by CYP2D6 metabolic activity. TQ offers practical advantages over PQ, including a longer half-life and reduced dependence on *CYP2D6* metabolism.

### Summary

This study evaluated the impact of *CYP2D6* polymorphisms on the recurrence of *Plasmodium vivax* in patients treated with primaquine or tafenoquine. Results showed that CYP2D6 activity influenced recurrence in primaquine, but not in those treated with tafenoquine.
